# The Magneto–Mechanical Hyperelastic Property of Isotropic Magnetorheological Elastomers with Hybrid-Size Magnetic Particles

**DOI:** 10.3390/ma16237282

**Published:** 2023-11-23

**Authors:** Leizhi Wang, Ke Zhang, Zhaobo Chen

**Affiliations:** 1 School of Mechatronics Engineering, Harbin Institute of Technology, Harbin 150001, China; wangleizhi@hit.edu.cn; 2 School of Chemical Engineering and Technology, Harbin Institute of Technology, Harbin 150001, China

**Keywords:** magneto–mechanical compression, hyperelastic property, isotropic MRE, hybrid size, extended Knowles model

## Abstract

Isotropic magnetorheological elastomers (MREs) with hybrid-size particles are proposed to tailor the zero-field elastic modulus and the relative magnetorheological rate. The hyperelastic magneto–mechanical property of MREs with hybrid-size CIPs (carbonyl iron particles) was experimentally investigated under large strain, which showed differential hyperelastic mechanical behavior with different hybrid-size ratios. Quasi-static magneto–mechanical compression tests corresponding to MREs with different hybrid size ratios and mass fractions were performed to analyze the effects of hybrid size ratio, magnetic flux density, and CIP mass fraction on the magneto–mechanical properties. An extended Knowles magneto–mechanical hyperelastic model based on magnetic energy, coupling the magnetic interaction, is proposed to predict the influence of mass fraction, hybrid size ratio, and magnetic flux density on the magneto–mechanical properties of isotropic MRE. Comparing the experimental and predicted results, the proposed model can accurately evaluate the quasi-static compressive magneto–mechanical properties, which show that the predicted mean square deviations of the magneto–mechanical constitutive curves for different mass fractions are all in the range of 0.9–1. The results demonstrate that the proposed hyperelastic magneto–mechanical model, evaluating the magneto–mechanical properties of isotropic MREs with hybrid-size CIPs, has a significant stress–strain relationship. The proposed model is important for the characterization of magneto–mechanical properties of MRE-based smart devices.

## 1. Introduction

The magnetorheological elastomers (MREs) are magnetic field-driven smart composites whose main components are micro- and nano-sized magnetic particles, an elastic polymer matrix, and additives. Precursors are mixed and cured to form a three-dimensional cross-linked network between adjacent magnetic particles, resulting in magneto-responsive mechanical properties. The magnetically controlled properties of MREs are based on the interaction between the induced magnetic particles to generate magnetic moments. MREs exhibit magneto–mechanical coupling properties under an external magnetic field, with the advantage of a fast response and continuously adjustable characteristics. MRE matrices are generally made of rubber-like materials containing natural rubber [1], silicone rubber [2], thermoplastic rubber [3], synthetic rubber [4], etc., which have remarkable hyperelastic mechanical properties. Hyperelasticity is a generalization of linear elasticity that is non-linear and suitable for predicting large strains. The magnetic particles are the main condition that determines the magnetorheological effect of the MRE magneto–mechanical coupling. A comprehensive overview of the current state of research on MRE preparation, mechanical properties, and device applications [5] describes the generation of magnetorheological effects from microscopic and phenomenological perspectives. The elastic mechanical properties mainly depend on the polymer matrix and the magnetic field response associated with the magnetic filler. MREs can be used to design different types of actuators [6,7,8], vibration absorbers [9,10,11], vibration isolators [12,13,14,15], magneto–mechanical metamaterials [16,17,18], and acoustic metamaterials [19,20,21,22]. The magneto–hyperelastic mechanical properties of MREs have been extensively studied to provide a mechanical basis for developing related devices.

Different types of modeling approaches for magneto–mechanical coupling [23] in MRE include macroscopic continuum-based models, microstructure-based models, and data-driven phenomenological models. A microscopic modeling study based on the structure of magnetic dipoles or magnetic chains facilitates the analysis of the magnetoelastic mechanical behavior and reveals the non-linear magneto–mechanical coupling mechanism. A homogenized mechanical framework for magneto–elastic materials has been proposed, taking into account the effects of magnetic dipole interaction and finite strains [24], where the magneto–elastic energy is expressed as a purely mechanical part and a magneto-induced part in a deformed state using a partial decoupling approximation. Furthermore, the problem of stability of MREs undergoing finite deformation in the presence of a magnetic field was investigated [25], and general conditions for the occurrence of macroscopic instability were derived, focusing on the anisotropic chain-like structure into a multilayer structure. The deformation mechanisms of the microstructure of magnetic particles in the matrix are investigated by considering both magnetic and mechanical loading conditions [26], only the existing eight-particle cubic lattice, and constructing a hyperelastic constitutive model based on the strain energy density. The magnetoelastic mechanical behavior of a MRE with a magnetic chain microstructure under large deformation [27] was investigated using a microscopic magneto–mechanical coupling model to reveal the magneto–hyperelastic properties. Using the finite element method, a three-dimensional representative volume element was constructed to analyze the macroscopic stress and magnetic volume force properties. The magneto–mechanical properties of magneto-active elastomers [28] have been investigated from two different modeling perspectives: macroscopic continuum mechanics models and magnetic dipole microstructure modeling approaches, and comparative results show a clear agreement between these two modeling approaches. Due to the complexity of the microstructural modeling considering magnetic dipoles or chains, the continuum mechanical model of MREs can be generalized by simplifying the microstructural parameters but still taking into account the effects of magneto–mechanical coupling effects and microstructural deformations. The theoretical basis of the magnetoelastic response of multi-physical fields coupling [29] was investigated by proposing a constitutive equation of isotropic MREs within the framework of electromechanical and thermomechanical theories, which was applied in the mechanics modeling of cylindrical tubes under axial shear and radial magnetic field. A simplified finite-strain continuum mechanics model is established, taking into account the magneto–mechanical coupling effect and the deformation of the magnetic chain microstructure to facilitate the identification of material parameters [30]. The predictive ability of the proposed model was validated using the experimental data on the mechanics of isotropic and anisotropic MREs.

The hyperelastic mechanical modeling of MREs generally requires the identification of model parameters using the results of basic material mechanics experiments; therefore, the experimental procedure setting and the analysis of the mechanical behavior of MREs under an external magnetic field are very important parts. Isotropic and anisotropic MREs were characterized for quasi-static magneto–hyperelastic mechanical properties [31], depending on the microscale modeling based on different magnetic particle lattice structures and the non-linear hyperelastic mechanical modeling based on the Neo–Hookean model and the first-order Ogden model. The quasi-static compressive properties of isotropic MREs under a vertical magnetic field were experimentally characterized [32], and the Ogden hyperelastic model based on the experimental results can accurately predict the compressive hyperelastic behavior. The non-linear magnetoelastic coupling behavior of MREs [33] was investigated using the coupled Yeoh hyperelastic mathematical model to analyze the hyperelastic features through experimental characterization. Using the triaxial compression experimental apparatus, the hyperelastic mechanical properties of MREs are systematically studied for analysis of the elastic modulus, bulk compressive modulus, and Poisson’s ratio under different strain rates [34], which presents multi-stage states through the free compression stage, the transition stage, and the triaxial compression stage. The differences between the above models and those shown in this study are compared as follows. The parameters in the Yeoh model do not include the elastic modulus, leading to an unattainable term of the coupled magneto-induced elastic modulus, which is not considered in this study. The parameters of three models (the Neo–Hookean, the Ogden, and the Knowles models) fully include the shear modulus μ. However, both the Ogden and the Knowles models can be degenerated to the Neo–Hookean model when certain parameters are equal to one. Although both the Ogden and Knowles models have a parameter that regulates the hardening of the stress–strain curve, the form of the strain function in the Knowles model is more advantageous for analyzing the hyperelastic mechanical properties of particle-reinforced elastomers.

In this study, the isotropic MREs with different ratios and mass fractions of hybrid-size CIPs are prepared. The micro-morphology of these samples is characterized using an ultra-depth-field microscope. Meanwhile, the magnetization properties of the isotropic MRE with different hybrid size ratios and mass fractions were obtained via magnetic testing. Then, the magneto–mechanical hyperelastic properties of isotropic MREs are experimentally tested to analyze the effect of the hybrid size ratio, mass fraction, magnetic flux, and compression strain on the magneto–mechanical constitutive properties. An extended Knowles model based on magnetic energy was proposed to predict stress–strain laws under a magnetic field and compression mode. The experimental data are used to identify parameters of an extended Knowles model, which includes strain field-related parameters *b* and *n* and the magneto-induced modulus parameter *K*. Finally, the experimental results reveal the influence of the hybrid size ratio of CIPs on the magneto–mechanical hyperelastic properties of isotropic MREs. The magneto–mechanical hyperelastic properties of isotropic MRE were predicted using the proposed model comparing theoretical and experimental results.

## 2. Materials and Methods

**Figure 1:**
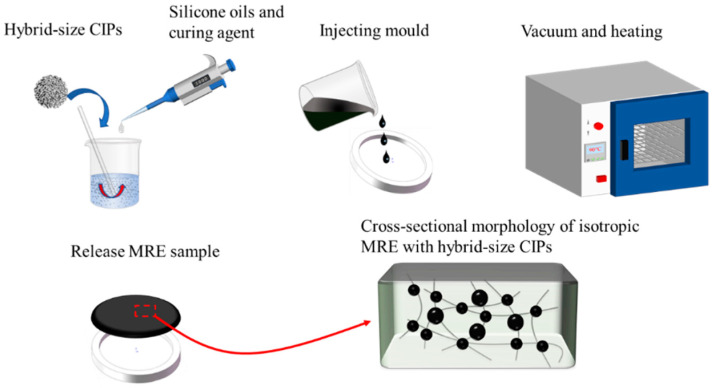
Illustration for the preparation process of MRE with hybrid-size CIPs.

The MREs of hybrid-sized magnetic particles were prepared from three raw components: carbonyl iron particles (CIPs), silicone rubber, and a curing agent. Two grades of CIPs were defined as CD and CN exhibiting particle size distribution (PSD) shown in Table 1, respectively, which were provided by BASF Co., Germany. The PSD of CIPs was tested using a laser particle size analyzer (Beckman LS 13320, B94600) provided by Beckman Coulter Inc., Indianapolis, IN, USA. The cumulative distribution percentages, including D10, D50, and D90, were calibrated to analyze CIP sizes shown in Table 1. The CIPs of the CN grade were larger than that of the CD grade in different percentages when comparing the particle size distributions of the two grades. The silicone rubber (KE-1606) and matching curing agent were supplied by Shin-Etsu Chemical Co., Tokyo, Japan. Silicone oil, provided by Dow Corning GmbH, Midland, MI, USA, was added to modulate the viscosity of the precursor to obtain a relatively low initial modulus. A higher relative magnetorheological effect can be achieved with the same mass fraction of magnetic particles. The preparation process of isotropic MRE specimens included the key steps shown in Figure 1: first, the two sizes of magnetic particles (40%, 60%, and 80% by mass) were weighed according to a specific CIPs ratio of CD and CN (1:3, 1:1, and 3:1), and then, the corresponding proportions of silicone rubber and silicone oil were weighed. Secondly, the precursor was mixed uniformly and poured into machined molds. It was then placed in a vacuum and heating chamber at 90 °C for 25 min to eliminate air bubbles in the mixture and cure to a solid. Finally, these MRE samples were removed from the molds and cut into standard shapes using a mold knife.

**Figure 2:**
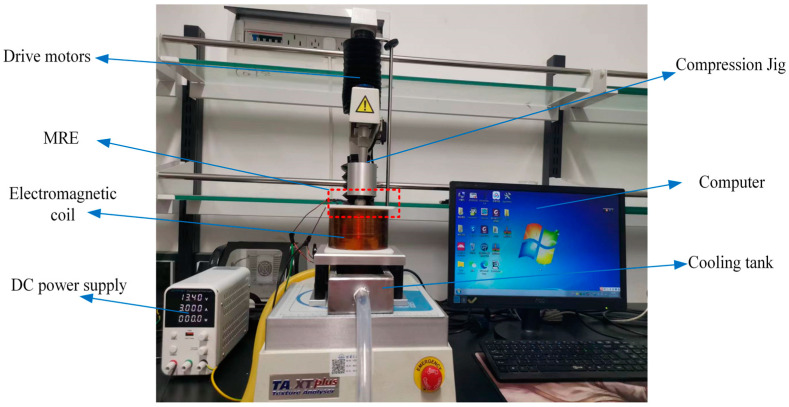
Experimental set-up for MRE magneto–mechanical compression tests.

The hyperelastic mechanical properties of the MREs with hybrid size CIPs under compression mode at room temperature were tested using a single-column texture analysis instrument (TA. XT Plus, Stable Micro Systems, Godalming, UK) equipped with an electromagnetic coil, as shown in Figure 2. The magnetic flux density, calibrated from the corresponding relationship of the control current and magnetic field, was measured using a digital Gauss meter, including a Hall sensor and microcontroller, while the MRE samples were placed in the gap. In order to avoid coil heating problems, the coil and core temperatures were maintained at room temperature during the prolonged test, using recirculated water cooling by reserving water in the cooling tank. The maximum magnetic field strength chosen was 215 mT to maintain a stable temperature of the solenoid coil’s iron core during long-term testing at a 6 A control current. A high-precision DC power supply was used to regulate the electromagnetic field in Figure 2. The compression test force accuracy can be up to 9.8 mN, and the drive motor’s maximum range is 490 N. Data were acquired from load to unload in each test to characterize the constitutive stress–strain properties of the specimens. The magnetic flux density was parallel to the direction of magnetization of the specimen. A factorial design of experiments was used with different levels of hybrid ratio (1:0, 1:3, 1:1, 3:1, and 0:1) and magnetic flux density (0, 81, 130, and 215 mT). Cylindrical specimens of the MRE were cut with a diameter of 10 mm and a thickness of 5 mm. The MREs with hybrid-size CIPs were tested according to the ASTM D-395-16 standard [35] for compression of rubber-like materials. The force measured in magneto–mechanical experiments was calibrated via the magnetic force between the MRE and the magnetic pole of the electromagnet. The force in the load cell consists of two components: the viscoelastic force-induced compression of the MRE specimens and the magnetic force-induced attraction of the magnetic pole. The direction of the magnetic force was opposite to the direction of the viscoelastic force, so it was necessary to subtract the magnetic force from the total cell force.

## 3. Results and Discussion

### 3.1. Microstructure of MREs

**Figure 3:**
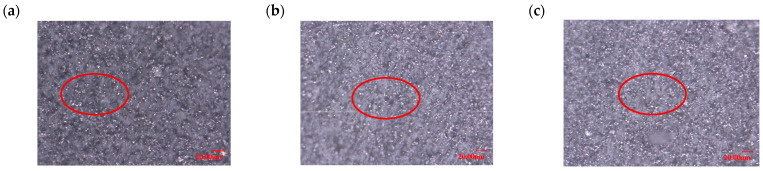
Cross-section microscopic morphology of isotropic MRE with 60% mass fraction at different ratios of hybrid size CIPs (CD:CN): (**a**) 1:3; (**b**) 1:1; and (**c**) 3:1.

The microstructures of the MRE sample were observed using an ultra-depth-field microscope with a resolution of 20 μm, showing the cross-sectional morphology of MRE with different ratios of hybrid-sized CIPs. Due to the metallic nature of the magnetic particles, they appear as white dots in the image, while the black background is the silicone rubber matrix. The change in the microscopic morphological features is that the number of magnetic particles per unit area of the cross-section gradually increases as the ratio of large-sized magnetic particles decreases, as shown by the red circles presenting the same unit area in Figure 3a–c.

### 3.2. Magnetic Properties of Hybrid-Sized CIPs in the MREs

**Figure 4:**
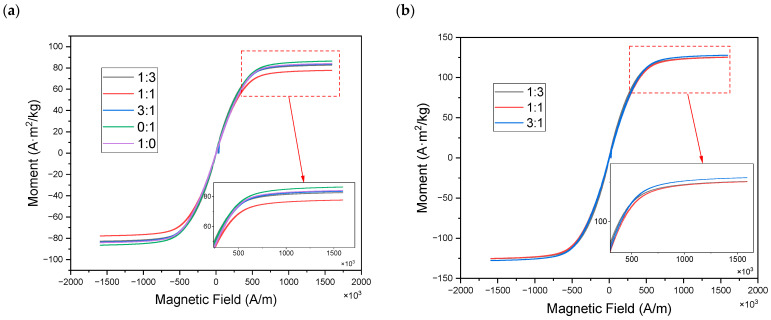
Magnetic hysteresis loop of isotropic MRE with different hybrid size ratios (CD:CN): (**a**) 40% mass fraction CIPs and (**b**) 60% mass fraction CIPs.

**Figure 5:**
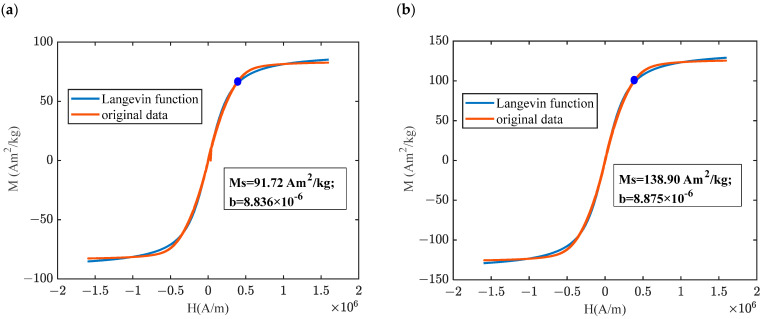
Comparison of theoretical and experimental results in magnetization of different mass fractions: (**a**) 40% and 1:3 and (**b**) 60% and 1:3 (CD:CN).

The magnetic properties of MREs with hybrid-size CIPs were characterized using a vibrating sample magnetometer (VSM), as shown in Figure 4. The VSM device mainly consists of an electromagnet system, sample forced vibration system, and signal detection system. It can be seen that CIPs exhibit a classical soft magnetic property with a low coercive force, residual magnetization, and high saturation magnetization. Hysteresis loops of isotropic MREs for different ratios and mass fractions all exhibit essentially coincident reciprocal paths; however, the saturation magnetization trend is affected by the different size ratios within the MREs regardless of the mass fractions in Figure 4a,b. The results show that the higher the ratio differentiation, the higher the magnetic saturation. Quantitative analysis of the magnetization of CIPs inside the MRE was performed using the Langevin function ($M=M_{s}L\left(bH\right)=M_{s}\left[\coth\left(bH\right)-1/\left(bH\right)\right]$) under different magnetic fields (*H*), where *M_s_* is the saturation magnetization and *b* is the shape parameter of the curve. Therefore, different ratios of hybrid-sized CIPs represented parameters of the fitted curves for the variability. For example, the Langevin functions of the magnetization of isotropic MREs with different mass fractions at a ratio of 1:3 are given as shown in Figure 5, which showed that the linear magnetization trend and the accurate description of function in the range below 3.964 × 10^5^ A/m, marked with a blue dot, compared to the higher magnetic field ranges.

### 3.3. Magneto–Hyperelastic Mechanical Model

The compressive magneto–hyperelastic behavior of MREs under different magnetic fluxes and strains was tested experimentally by describing the stress–strain curves. The experimental results were used to identify the parameters of extended Knowles hyperelastic models, considering the magneto–mechanical coupling effect. The magneto–hyperelastic mechanical model is an extension of the Knowles model that can improve the accuracy of the Helmholtz free energy per unit reference volume [36] under the compression behavior in Equation (1).

(1) $$E\left(\bar{I_{1}},J\right)=-\frac{\mu }{2b}\left\{{\left[1+\frac{b}{n}\left(\bar{I_{1}}-3\right)\right]}^{n}-1\right\}+\frac{\kappa }{2}{\left(J-1\right)}^{2},$$

where *μ* is a shear modulus; *n* is a stiffening (*n* < 0) or softening (*n* > 0) parameter; b is a shape parameter; and *κ* is a bulk modulus. MREs are particle-reinforced polymer composites with apparent strain-hardening effects. Therefore, the parameters in the hyperelastic framework characterize the trend of the constitutive curve of MRE with hybrid-size CIPs. *I*_1_ is the first invariant, and the equation for free energy is linear in *I*_1_. *J* is the determinant of the deformation gradient. The energy from volumetric deformations is quadratic in (*J* − 1), giving a volumetric stress that is linear in (*J* − 1), shown in Equation (2). The elastic deformation tensor is the matrix $\bar{B}$ and the unit matrix is denoted as *I*. In particular, the special form when both *n* and *b* are one corresponds to the Neo–Hookean model. For the incompressible Knowles model, the Cauchy stresses in uniaxial deformation are shown in Equation (2).

(2) $$\sigma =\frac{\mu }{J}{\left(1+\frac{b}{n}\left(\bar{I_{1}}-3\right)\right)}^{n-1}\left(\bar{B}-\frac{1}{3}\bar{I_{1}}I\right)+\kappa \left(J-1\right)I.$$

In principle, the local magnetic forces inside MRE are represented as the negative gradient field of the magnetic energy [28]. The magnetic energy of a microsphere is calculated in the form of finite volume integration. The magnetic dipoles approach is usually used to build magnetic free energy relating microstructure parameters [37], such as the radius of the particle *a*, position vector *r*, and the angle *θ* between the direction of magnetic field H_0_ and the position vector ***r***. For the mechanical modeling of anisotropic MRE, the magnetic dipoles approach can characterize the direction of polarization of the magnetic chains to analyze magneto-induced mechanical properties. This can help us understand the action mechanism of magnetic dipoles in isotropic MRE. However, there is not an obvious major polarization direction for the homogenized MRE, thus simplifying the magnetic free energy calculation instead of the magnetic dipoles approach. The magnetic free energy *U* of CIPs in isotropic MRE as a function of the strain *ε*, which can be expressed in Equation (3) [38,39]. The main parameter of the magnetic free energy is the magnetization per unit mass *M*, where is depending on the volume fraction ϕ of CIPs. *χ*_0_ is the permeability of the vacuum, and *M_s_* is the saturation magnetization.

(3) $$U\left(\varepsilon \right)=\frac{\chi_{0}M_{s}^{2}}{4\pi }{\left(\phi M\right)}^{2}f\left(\varepsilon \right),$$

A magnetization function, as shown in Equation (4), was used to calculate the magneto-induced elastic stress. *ρ* is the MRE material density, and *H* is the magnetic field strength. Ms is the saturation magnetization, and *b* is a shape parameter fitted by the results of magnetic characterization. *M_MRE_* is the total magnetization of the isotropic MRE.

(4) $$M_{MRE}={\left(M\rho \right)}^{2}=M_{s}{}^{2}\rho^{2}{\left[\coth\left(bH\right)-\frac{1}{bH}\right]}^{2},$$

The function of the magneto-induced elastic stress $\Delta \sigma_{m}$ is the first derivative expression of the magnetic free energy with respect to the compressive strain, which is expressed in Equation (5) as a strain function *f*(*ε*) as follows.

(5) $$\Delta \sigma_{m}=\frac{\chi_{0}M_{s}^{2}}{4\pi }\frac{\partial f\left(\varepsilon \right)}{\partial \varepsilon }M_{MRE}^{2}\left(H\right).$$

Due to the linear relationship between the elastic modulus and shear modulus for isotropic MRE, the increase in shear modulus in the Knowles model can also be assumed to have a square relationship with the magnetization coefficient in Equation (6), where *K* is the material constant, which presents $\chi_{0}M_{s}^{2}/\left(4\pi \right)$ and is independent of the compressive strain for simplicity. Therefore, *μ* is an elastic parameter that depends on the magnetic field-dependent function shown in Equation (7), where *μ*^0^ is the Knowles model shear modulus at a zero magnetic field.

(6) $$\Delta \mu_{m}\left(H\right)=KM_{MRE}^{2}\left(H\right),$$

(7) $$\mu \left(H\right)=\mu^{0}+KM_{MRE}^{2}.$$

Substituting Equation (7) into the Knowles model, the magneto–hyperelastic constitutive model is finally presented in Equation (8), where *K* is the identified parameter of the magneto-induced modulus, the unit of which is expressed as J/(A^2^·m). λ is a true strain in compression. It is multiplied by the square of the MRE magnetization to obtain the Helmholtz free energy per unit volume (J/m^3^).

(8) $$\sigma_{uniax}=\left(\mu^{0}+KM_{MRE}^{2}\right){\left[1+\frac{b}{n}\left(\lambda^{2}+\frac{2}{\lambda }-3\right)\right]}^{n-1}[\lambda^{2}-\frac{1}{\lambda }].$$

### 3.4. Experimental Result of Magneto–Elastic Mechanics

The measured stress–strain properties of the MRE with hybrid-size CIPs revealed hyperelastic properties during the loading and unloading process. The non-linear stress characteristics for different magnetic flux densities were experimentally analyzed to determine the Knowles magneto–hyperelastic model as functions of the volume fraction of magnetic particles, magnetic flux density, strain, and material elastic parameters. Therefore, the results show a strong dependence of the elastic modulus on strain level and direction of load change and only a dependence of the magneto-induced modulus on magnetic flux and CIP mass fraction. In the following, the magneto–hyperelastic properties of MRE with different ratios of hybrid CIPs are analyzed to obtain the effect of the ratio coefficients on the constitutive properties. The phenomenon of parameter effects was revealed using the MRE magneto–mechanical coupling test under 10% strain considering five mixing ratios, four magnetic field levels, and two mass fractions. In previous studies, the strain amplitude significantly affects the stress–strain characteristic curves, presenting strain-softening phenomena [40], independent of both static and dynamic mechanical behavior. Comparable quasi-static hysteretic stress–strain properties were obtained for isotropic MRE with hybrid-size CIPs, which exhibit similar dependence on the ratio of two CIP sizes. Meanwhile, the mass fraction of CIPs in the MRE is a significant parameter for the initial elastic modulus and the magneto-induced elastic modulus.

**Figure 6:**
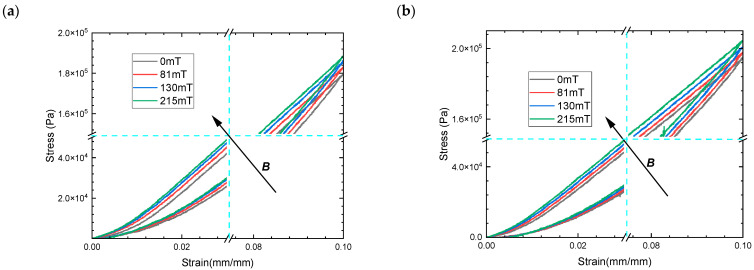
The magneto–mechanical experimental results of MREs with 40% mass fraction compressive mode (**a**) CIPs of CD grade corresponding to ratio 1:0, and (**b**) CIPs of CN grade corresponding to ratio 0:1.

The quasi-static compression test of MRE with single-size CIPs under a magnetic field was first carried out as a control to analyze the influence of hybrid-size CIPs on the magneto–elastic mechanical properties of MRE. The results show that the peak stress of a MRE with large-size CIPs is significantly higher than that of a MRE with small-size CIPs, shown in Figure 6 for the same mass fraction. However, the size of the magnetic particle has no significant effect on the magneto-induced stress at the same mass fraction. The direction of arrow B represents a gradual increase in the magnetic flux density of the compression test. The induced magnetic field in the compression experiment is an external spatial magnetic field, which induces the generation of magnetization-free energy inside the MRE. Based on the trend of the magnetization curves, it is also verified that isotropic MRE with different hybrid size ratios has the same unit magnetization energy when below 488 mT flux density. This can be attributed to having the same total magneto-induced energy of isotropic MREs exhibiting directional magneto-induced stress. The constitutive curves show more obvious nonlinearity within small strains as magnetic flux density increases, which suggests a gradual reduction in non-linear trends for MRE of different-size CIPs. The reason for the weakening of the non-linear trend is the hardening effect of MRE under a magnetic field, where the rate of change of the elastic modulus decreases and stabilizes with increasing strain.

**Figure 7:**
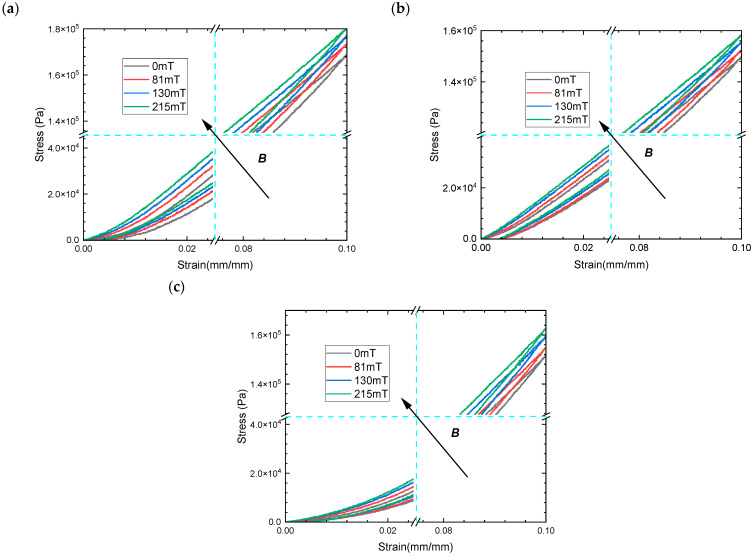
The magneto–mechanical experimental results of MREs with hybrid-sized CIPs (CD:CN) of 40% mass fraction under compressive mode (**a**) stress–strain curves of MRE with the weight per cent 1:3; (**b**) the weight per cent 1:1; and (**c**) the weight per cent 3:1.

The comparable trends of the stress–strain curves as a function of the ratio of hybrid size CIPs show the strong non-linear features of the material in the less than 2% strain range as the ratio of small size CIPs increases at the same mass fraction in Figure 7a–c. This is due to the higher local stress within the MRE, which represents interactions between small and large magnetic particles. Thus, the phenomenon is more evident at small strains, while the constitutive load curve tends to be linearized at 10% of large strains at the loading path. These also cause a greater asymmetry in the stress–strain characteristics even at the higher strain of 10%. In particular, the elastic modulus of isotropic MRE decreases as the proportion of small-sized magnetic particles increases despite the same mass fraction of magnetic particles. In essence, MREs are particle-reinforced polymer composites, a phenomenon that contradicts the conventional view that particles of the same mass fraction have the same modulus of elasticity. Meanwhile, Figure 7 illustrates the effect of magnetic flux density, ranging from B = 0 mT to B = 215 mT, on the stress–strain response of isotropic MREs with hybrid-size CIPs subjected to a 10% compressive strain. The results show that the axial slope of the strain path increases with increasing magnetic flux intensities, which have essentially the same growth range. The maximum magneto-induced elastic modulus is 0.11 MPa under a 10% strain in the above magnetic field interval. This is because the main controlling factor of the magnetic energy generated via the MRE magnetization is the mass fraction of the CIPs, which is independent of particle size. 

**Figure 8:**
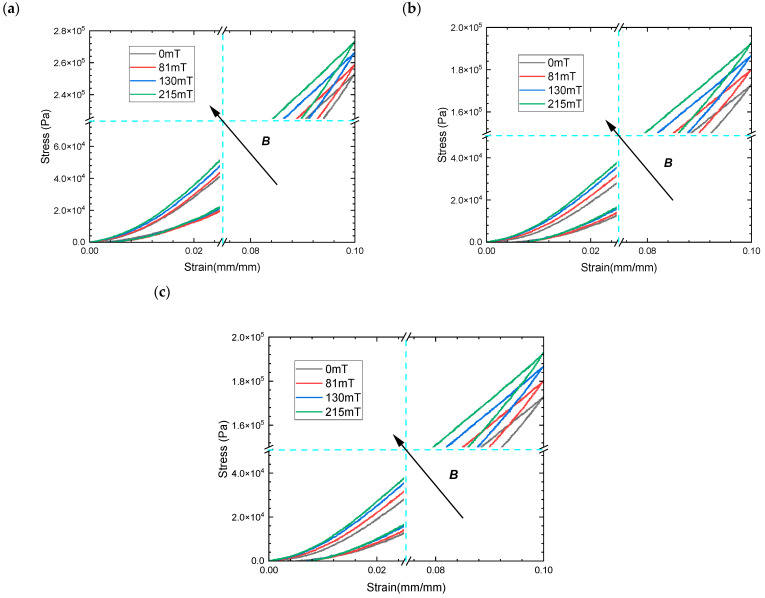
The magneto–mechanical experimental results of MREs with hybrid-sized CIPs (CD:CN) of 60% mass fraction under compressive mode (**a**) stress–strain curves of MRE with the weight per cent 1:3; (**b**) the weight per cent 1:1; and (**c**) the weight per cent 3:1.

In addition, to compare and validate the above phenomena, the magneto–mechanical coupling hyperelastic properties of the isotropic MREs with hybrid size CIPs of 60% mass fraction were analyzed, as shown in Figure 8. The larger mass fraction MRE gives significantly higher peak stress and magneto-induced stress during loading for the same compressive strain and magnetic flux density. In particular, the non-linear characteristics of the higher mass fraction MRE stress–strain curves are more pronounced at small strain states, which present a gradual stiffening phenomenon of the slopes as the magnetic flux density increases. This is due to a relatively higher mass fraction of CIPs in isotropic MRE, where more closely spaced particles under compressive behavior contribute to a non-linear increase in the elastic stress under compressive behavior. The magneto-stiffening effect is more significant for MREs with a larger proportion of CIPs due to a higher magneto-induced elastic modulus. This increase in the zero-field elastic stress inside MREs at 10% strain becomes more pronounced with an increase in the ratio of large-size CIPs, from 25% to 75% at the same mass fraction, which shows an increase of up to 46.67%, resulting in greater stiffening during loading as shown in Figure 8a,c. However, the magneto-induced stress or elastic modulus has not changed as the ratio of larger-size CIPs increases at the same magnetic flux density, showing an elastic modulus increase of 0.202 MPa at 215 mT.

**Figure 9:**
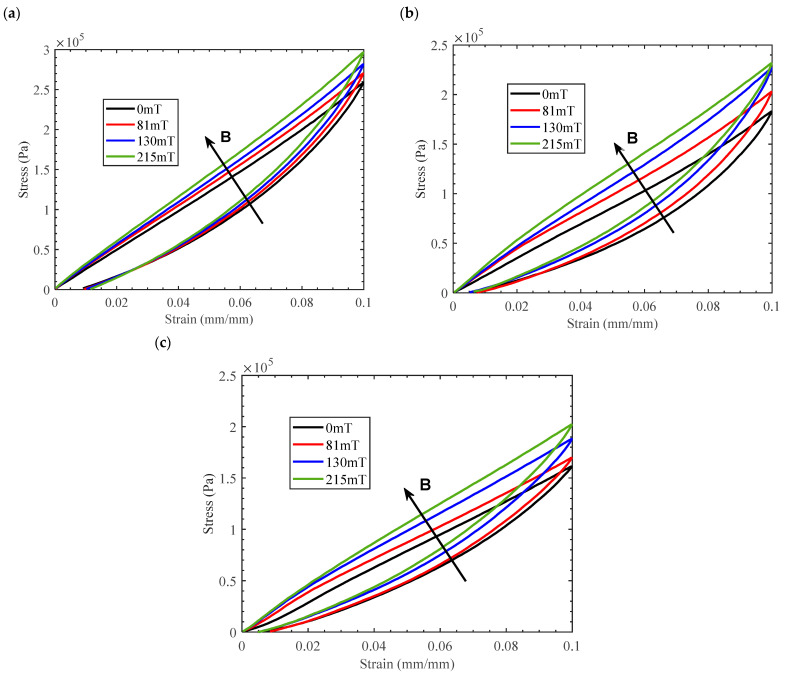
The magneto–mechanical experimental results of MREs with hybrid-sized CIPs (CD:CN) of 80% mass fraction under compressive mode (**a**) stress–strain curves of MRE with the weight per cent 1:3; (**b**) the weight per cent 1:1; and (**c**) the weight per cent 3:1.

Furthermore, the magneto–mechanical constitutive properties of MRE with an 80% mass fraction of CIPs were analyzed using the results of compression experiments in Figure 9. The results reveal that the hybrid size ratio of high-content magnetic particles affects the magnetic stress–strain curves of MRE. The zero-field elastic modulus decreases with the gradual increase in the ratio of small-size particles, which is consistent with that of MREs above 40% and 60% mass fraction. The magneto-induced stress of MRE increases by about 40 KPa for all hybrid size ratios in Figure 9a–c as the magnetic flux density increases from zero to 215 mT, corresponding to a maximum strain of 10%.

**Figure 10:**
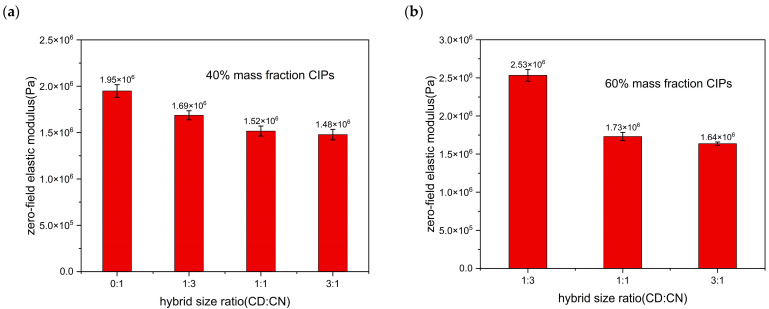
Zero-field elastic modulus of an isotropic MRE at different ratios of hybrid size CIPs: (**a**) 40% mass fraction and (**b**) 60% mass fraction.

A higher proportion of large-size CIPs exhibits a higher zero-field elastic modulus of isotropic MRE at both 40% and 60% mass fractions shown in Figure 10a,b. The experimental error bar of the zero-field modulus is given a fluctuating range in Figure 10, showing a smaller error range of the zero-field modulus of MRE with a 60% mass fraction than that of a 40% mass fraction. It is necessary to consider the enhancement effect of filled particles within a polymer matrix under compressive behavior, which reveals that particle size selection can effectively regulate the zero-field modulus of a MRE. The mechanism is that the surface area of the magnetic particles in contact with the matrix decreases inside the elastomer, resulting in an increase in local stress. Thus, the zero-field modulus changes abruptly, violating the Einstein–Guth–Gold equation [41]. This method facilitates broadening the relative magnetorheological rate of MREs used in vibration absorbers or isolators to achieve a high bandwidth of vibration suppression. For a MRE with higher mass fraction CIPs, the effect of the hybrid size ratio on the zero-field modulus is significant. 

**Figure 11:**
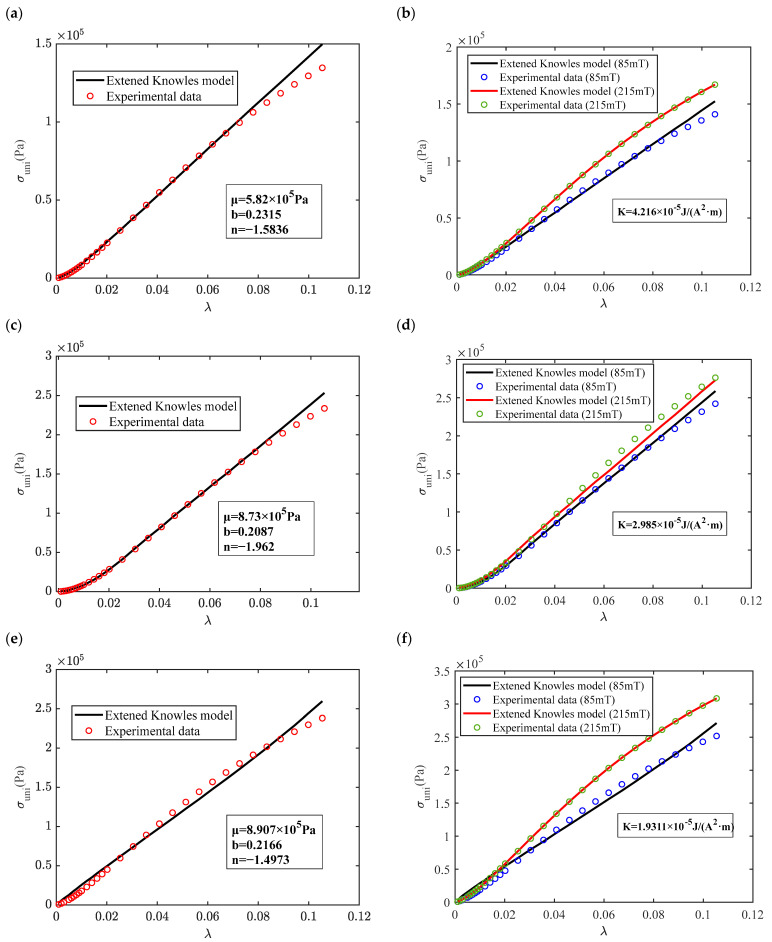
Comparison of magneto–mechanical stress–strain curves of an isotropic MRE with a hybrid size ratio of 1:3 (CD:CN) using the results of an extended Knowles model and experimental test: (**a**) zero-field stress–strain curve (40% mass fraction CIPs); (**b**) stress–strain curves under different magnetic fluxes (40% mass fraction); (**c**) zero-field stress–strain curve (60% mass fraction); (**d**) stress–strain curves under different magnetic fluxes (60% mass fraction); (**e**) zero-field stress–strain curve (80% mass fraction); and (**f**) stress–strain curves under different magnetic fluxes (80% mass fraction).

**Figure 12:**
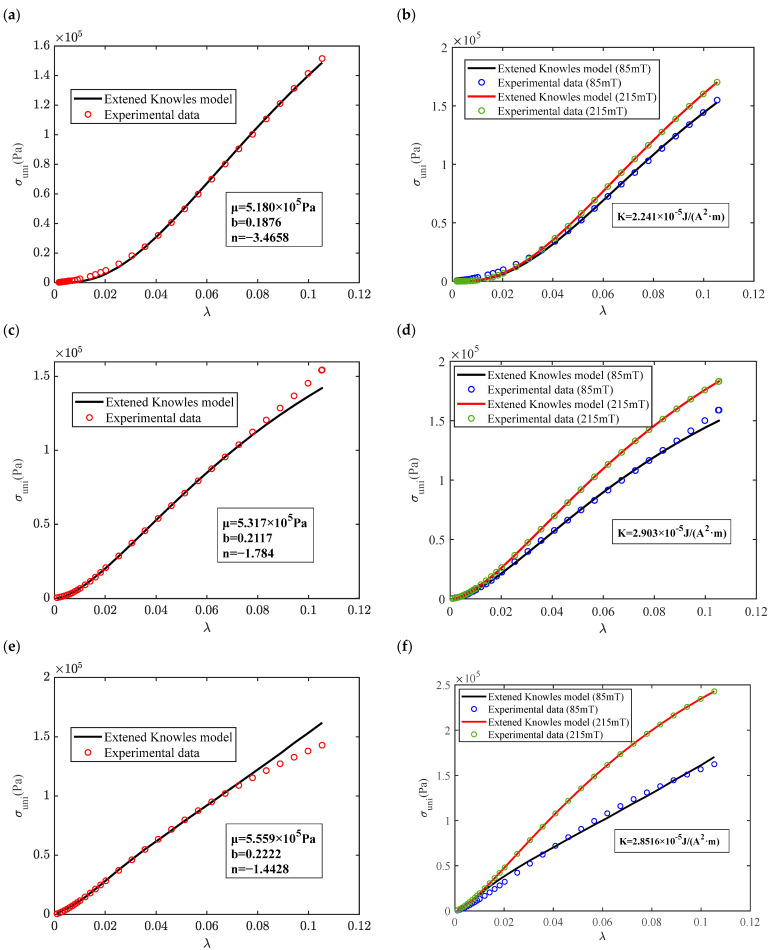
Comparison of magneto–mechanical stress–strain curves of an isotropic MRE with a hybrid size ratio of 3:1 (CD: CN) using the results of an extended Knowles model and experimental test: (**a**) zero-field stress–strain curve (40% mass fraction CIPs); (**b**) stress–strain curves under different magnetic fluxes (40% mass fraction); (**c**) zero-field stress–strain curve (60% mass fraction); (**d**) stress–strain curves under different magnetic fluxes (60% mass fraction); (**e**) zero-field stress–strain curve (80% mass fraction); and (**f**) stress–strain curves under different magnetic fluxes (80% mass fraction).

The main factor that determines the isotropic magneto-induced elastic modulus is the mass fraction of the magnetic particles independent of the magnetic particle size through the results of magneto–mechanical tests. Therefore, the identification of the parameters of the proposed model, corresponding to the magneto–mechanical hyperelastic characteristic, was carried out using an isotropic MRE with hybrid size ratios (1:3 and 3:1) of 40%, 60%, and 80% mass fraction CIPs, as shown in Figure 11 and Figure 12, as comparative examples. The experimental and theoretical results of the loading process at different magnetic fluxes were analyzed using an extended Knowles model. The unknown parameters in the magneto–hyperelastic model are identified using the magnetization corresponding to the response magnetic field in the *M*-*H* curve. The MRE constitutive function under the zero magnetic field is first determined to obtain the shape parameter *b* and the hardening parameter *n*. In particular, the elastic modulus of an isotropic MRE is calculated from the experimental results, and the shear modulus *μ* is obtained using the equation G = E/2(1 + υ). As shown in Figure 11a,c,e, the Knowles model has superior accuracy in describing the stress–strain relationship for the zero-field constitutive curves of MRE with a hybrid size ratio (1:3) at different mass fractions of CIPs. The identification accuracy of the hyperelastic properties of a MRE with different mass fractions is shown in Figure 11b,d,f. However, the model predictions for high-strain intervals greater than 8% did not perform as well as the predictions for low-strain intervals. The coefficients *n* are all less than zero, indicating a softening effect, while the shape parameters *b* are all less than one, corresponding to the non-linear variation of the shape of the constitutive curve under a small deformation compared to the Neo–Hookean model (*n* = 1 and *b* = 1). The theoretical curves of the magneto–mechanical model at different flux densities are obtained by substituting the identified parameters *b* and *n* into Equation (8), which in turn identifies the magneto-induced modulus coefficient *K* shown in Figure 11b,d,e, respectively. Since the density of the isotropic MRE is included in the proposed theoretical model, the densities of the MRE with 40%, 60%, and 80% mass fractions were measured to be 1430.104 kg/m^3^, 1803.16 kg/m^3,^ and 2887.32 kg/m^3^, respectively. The analysis results show that the magneto-induced modulus coefficient *K* decreases with increasing mass fraction. And the magneto–mechanical constitutive curves of MREs at different mass fractions represent a non-linear trend with mean squared coefficients of 0.9889, 0.9289, and 0.9627, respectively. The proposed magneto–hyperelastic model of an isotropic MRE is used to describe the stress–strain relationship for large deformation, which is correlated with the zero-field shear modulus and the magnetization coefficient.

In turn, the magneto–mechanical constitutive properties of MREs with a hybrid size ratio of 3:1 are predicted using an extended Knowles model representing a high proportion of small magnetic particles. For both zero-field and magneto–mechanical constitutive properties, this model predicts the 40% mass fraction MRE to be more accurate than the 60% and 80% mass fractions in Figure 12. Zero-field constitutive properties of MREs with the 60% and 80% mass fractions are predicted with lower accuracy at high strain intervals greater than 8% strain compared to the low-strain interval. The hardening parameter n of MRE with the 40% mass fraction MRE is smaller than that of the large mass fraction MRE, which also verifies the change in the slope of the constitutive curves in the experimental results in Figure 12a,c,e. And the shape parameter b varies around 0.2 to correct the shape of the constitutive curve. The identification of the magneto-induced modulus coefficients using the proposed model is achieved to analyze the theoretical and experimental values of the magneto–mechanical constitutive curves in Figure 12b,d,f for different mass fractions of MREs with a hybrid size ratio of 3:1. The results present that the mean squared coefficients are all in the range of 0.9–1.

## 4. Conclusions

In this study, the influence of the hybrid size ratio on the magneto–mechanical hyperelastic properties of MREs was experimentally investigated to use validation of an extended Knowles model that incorporates a magneto–elastic energy component. The results showed that the zero-field elastic modulus increased with an increasing ratio of larger-size CIPs at the same mass fraction. However, the magneto-induced elastic modulus did not change with the variation of the ratio depending on the total magnetic particle mass fraction. For the higher ratio of large-size CIPs, the non-linear characteristic under small strain showed magneto-induced stiffening phenomena, which became more significant as the magnetic flux density increased.

The Knowles constitutive model was combined with the magneto-induced stress function in Equation (5), depending on the magnetic parameter and strain function, to develop an extended Knowles model. By analyzing the magneto–mechanical hyperelastic properties of an isotropic MRE with hybrid size CIPs, the constitutive model was identified to predict the relationship between the elastic modulus and magnetic flux densities. Comparing the theoretical predictions and experimental results, it presented an accurate prediction of compressive stress–strain over the range of magnetic fields and strains. The predicted mean square deviation of the isotropic MRE magneto–mechanical constitutive curves is 0.9089 and 0.9889 for 40% and 60% mass fractions, respectively. For magneto–mechanical properties of isotropic MRE under a large strain, the proposed model can better describe the stress–strain law, which only needs to implement the identification of two zero-field parameters and one magnetic field parameter. In particular, a field-dependent parameter *K* that represents the dependence of the material magneto-induced modulus on the magnetization of an isotropic MRE is defined as the magneto-induced modulus coefficient. This model has a high potential to be applied to the magneto–mechanical characterization of MRE smart devices.

## Figures and Tables

**Table 1:** The particle size distribution of CD and CN grades originating BASF Co.

PSD	Unit	CD	CN	Test Method
D10	µm	2.0–3.3	3.0–4.0	Beckman LS 13320 (RCA/Q-C-300)
D50	µm	4.2–6.3	6.5–8.0	
D90	µm	7.5–12.0	14.0–27.0	

## Data Availability

All data used to support the findings of this study are available from the corresponding author upon request.
